# Vestibular migraine with Wallenberg syndrome: a case report

**DOI:** 10.1186/s12883-020-01786-1

**Published:** 2020-05-25

**Authors:** Ying Xin, Junyi Fu, Longchang Xie, Jie Yang, Guanhao Ke

**Affiliations:** 1 Department of Neurology, The 2nd Affiliated Hospital of Guangzhou Medical University, Guangzhou, 510260 Guangdong China; 2 Institute of Neuroscience and the Second Affiliated Hospital of Guangzhou Medical University, Key Laboratory of Neurogenetics and Channelopathies of Guangdong Province and the Ministry of Education of China, Guangzhou, 510260 China

**Keywords:** Vestibular migraine, Wallenberg syndrome, Vertigo, Headache, Case report

## Abstract

**Background:**

Vestibular migraine, a kind of acute vestibular syndrome, leads to both migraines and vertigo symptoms in a single patient. The occurrence of vestibular migraine has shown an obvious increase in female groups based on age. Though it is recognized that migraines may cause ischemic lesions in some brain regions, the relationship between vestibular migraine and cerebral infarction has seldom been reported, especially with no known research reports about vestibular migraine with Wallenberg syndrome. Based on this, the connection of the two diseases needs to be the focus of more research.

**Case presentation:**

The patient, a 35-year-old lady, came to our department with severe vertigo and headaches for approximately two years. She suffered from migraines which attacked about twice yearly for nearly a decade. The diffusive weighted imaging showed a subacute infarction in the right lateral medullar. The clinical characteristics and MRI findings supported the diagnosis of vestibular migraine with Wallenberg syndrome. Along with the normal routine medication for vestibular migraine with Wallenberg syndrome, we also prescribed migraine therapy at the same time. In a 3-month follow-up, the patient had suffered only one vertigo attack and she reported that the migraines were less common and less intense than she was previously experiencing.

**Conclusions:**

Due to the fact that vestibular migraine is one of the risk factors of cerebral ischemia, we need to pay more attention to this phenomenon. The current case suggests that both routine medication on ischemic stroke as well as treatment for migraines should be used concurrently in vestibular migraine with Wallenberg syndrome.

## Background

Vestibular Migraine (VM) is one of the most common diseases with vertigo as a symptom [1]. The diagnosis of VM is more and more accurate in recent years due to neurologists recognizing this disease [2–4]. Wallenberg syndrome includes a series of symptoms caused by lesions in medulla oblongata. It usually occurs in patients with obstruction of the vertebral artery, posterior inferior cerebellar artery (PICA) or lateral modularly arteries. So far there has been no report focusing on VM diagnosed concurrently with Wallenberg syndrome. In this case, we focus on a 35-year-old female patient, who suffers from recurrent VM and has also been diagnosed with Wallenberg syndrome. Due to the potential relationship between migraines and cerebral ischemia, this case is likely to reveal that early therapy for both ischemia and migraines in patients with Wallenberg syndrome caused by VM is an effective treatment.

## Case presentation

A patient, 35-year-old female, came to our clinic because of severe vertigo and paroxysmal headaches for about 2 years. She mostly suffered from vertigo at night with multiple vomiting spells and bilateral tinnitus, which would last the entire night. During the period of vertigo, she also had a headache at the right temporal site, which was present a kind of pulsatile pain and could last several hours; this caused nausea and the inability to fall asleep. Prior to the onset of the vertigo and headache, she also had a visual aura with wave sight that lasted 10 min. Approximately 10 of these attacks were trigged by loud noises or bright lights, accompanied with symptoms such as chest tightness, tachypnea, and blushing. The migraine attacks were mostly accompanied by vertigo, becoming more severe during vestibular episodes. Those symptoms continued to worsen over the next week from the initial onset. During this time, the patient suffered from vertigo for several hours daily, and also suffered from repeated vomiting, numbness on the right side of the face, and tinnitus in the ear. The attacks of vertigo had no connection to changing body position. The headache occurred on the right side with visual aura expressing as fortification spectrum during vertigo. Physical examination found dysesthesia around the right side of the forehead and unsteady gait.

The patient’s clinical history revealed a more frequent occurrence of migraines during the period of pregnancy. The headache was usually a throbbing, unilateral temporal pain for 20 min each time. It would result in nausea and vomiting, which led to functional limitation in daily activities and led to bed rest to alleviate her symptoms. Meanwhile, the patient also had a visual aura with waves of light that lasting approximately 10 min. She had no family history regarding her illness, history of drug use, allergy, smoking, or drinking.

A neurological examination showed clockwise rotary nystagmus when she gazed to the left side, and an abnormal finger-to-nose test at the right side. The patient had normal muscle tone and muscle strength, and no appearance of the Babinski Sign. Vestibular system tests including Dix-hallpike, Roll-test as well as a head thrust test were all negative as well.

Laboratory test showed the HbA1C was 5.1%, and plasma homocysteine was 9.5 μmol/L. The autoimmune antibodies including pANCA、cANCA(−)、MPO、PR3、ENA、ACA-IgA、ACA-IgG、ACA-IgM、ANA、ds-DNA、DNP were negative except for the AECA(++). Thrombophilia markers were also tested, including protein C activity (130%)、von Willebrand factor activity (109%) 、free protein S activity (76%) as well as antithrombin III activity (109%). Other tumor markers regarding lung cancer and colon carcinoma were all negative and thyroid gland function was also normal. The routine test of cerebrospinal fluid (CSF) was normal, while the aquaporin (AQP) 4 antibodies were negative in both plasma and CSF.

**Fig. 1:**
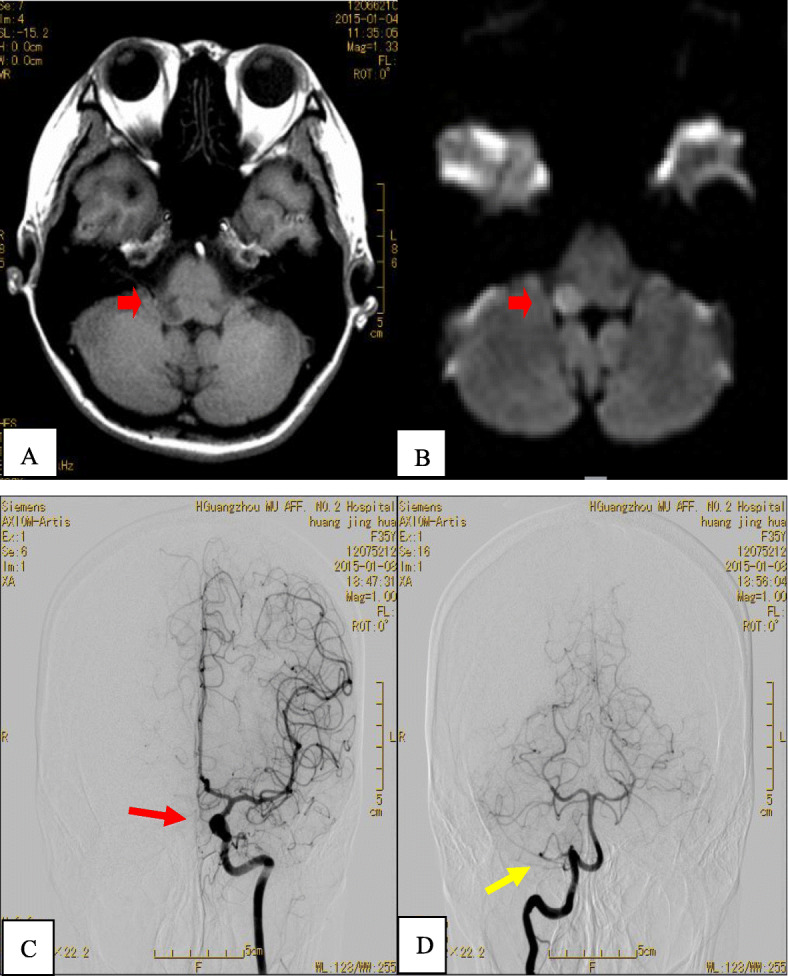
T1-weighted Magnetic resonance imaging (**a**) and diffusion-weighted imaging (**b**) showed subacute infarction (red arrow) in the right lateral medullar. Digital subtraction cerebral angiography showed an aneurysm in C2 segment of left internal carotid artery, about 6 mm × 5 mm, with smooth edge (red arrow) (**c**). Right posterior inferior cerebella artery could be seen clearly on DSA (yellow arrow) (**d**)

The electro-audiometry test revealed a mild hearing impairment in the right ear. Other Neuro-electrophysiology tests, including brainstem auditory evoked potential (BAEP)、visual evoked potential (VEP)、electromyogram (EMG) as well as nerve conductive velocity (NCV) were all normal. The cardiac and carotid ultrasound exams were both negative. The diffusive weighted imaging showed subacute infarction in the right lateral medullar on January 8th (Fig. 1a, b). The digital subtraction cerebral angiography (DSA) indicated a left intracranial aneurysm on the next day (Fig. 1c). The infarction and intracranial aneurysm were located on separate sides of the brain, which suggested that the Wallenberg syndrome might not be caused by the intracranial aneurysm. DSA imaging showed that the communicating artery between anterior and posterior circulation of cerebral arteries was not open, and ipsilateral posterior inferior cerebella artery could be seen clearly (Fig. 1d). We administrated antiplatelet treatment as well as Flunarizine immediately. The patient’s vertigo symptoms diminished just in a few days, with the patient being discharged three weeks later with oral medication, including Betahistine Mesilate 6 mg tid, Dihydroergotoxine 2.5 mg bid, Aspirin 100 mg qd and Flunarizine 5 mg qn. At the patient’s 3-month follow-up, she said she had only once been affected by vertigo and the migraines were less common and less intense than she was previously experiencing.

## Discussion and conclusions

The patient had acute onset of vertigo and right facial numbness accompanied by nausea, vomiting and an abnormal finger-to-nose test, which corresponded to all three of the following criteria: 1. the criteria of Wallenberg syndrome; 2.the criteria of vestibular migraine by International Headache Society (IHS) and Bárány Society [5]; 3. the criteria of migraineurs infarction by The International Classification of Headache Disorders 3rd Edition (ICHD III) [6].

VM is one of the most common vertigo diseases, the prevalence of which is approximately 1% [7]. It accounts for 11% of vertigo diagnosis, and has a five to one gender ratio between males and females. In females the average age for onset is 37.7 years, while it is 42.4 years in males [1]. The lifetime prevalence is estimated at 7% for vertigo and 16% for migraines; concurrent symptoms have been found in 3.2% in the entire population [8]. The pathophysiology of VM has not been clarified as of now, but some theories have shown a significant connection between the vestibular nuclei in the brainstem and the structures which could modulate the pathway of algesthesia conducted by the trigeminal nerve. One study using the functional magnetic resonance Imaging (fMRI) revealed that the dysfunction of vestibular-thalamic-cortical processing caused a dysmodulation of multimodal sensory integration, which might associate with the attacks of VM [9]. Moreover, it was reported that underlying genetic abnormalities had been found in vestibular migraines. The genetics of vestibular migraines are still under heterogeneous and are uncertain [10], some research reported that chromosome 5q35 was associated with VM. The predisposition factors of VM might include menses, irregular sleep, stress, exhaustion, dehydration, some food and drink, and drastic pain [11]. The treatment of VM has not been systematically studied, but the treatment process of migraines could include tricyclic antidepressants such as amitriptyline or selective serotonin reuptake inhibitors (SSRIs) and benzodiazepines as recommended [12]. Some reports showed that prophylactic medication might benefit the aura symptoms as well as unspecific migraine-related symptoms [13]. For example, topiramate demonstrated to have a confirmative effect on VM. The etiology of VM with Wallenberg syndrome is still unknown. However, both cortical spreading depressing (CSD) and trigemino-vascular theory were gradually accepted [14, 15]. The theory of CSD demonstrated that some adverse stimuluses triggered the inhibition bands of brain electrical activity from posterior cerebral cortex. The bands spread out in a wave (2-5 mm/min) slowly to the adjacent cortex, accompanied with “spreading depressing” cerebral blood flow. Trigemino-vascular theory demonstrated that the stimulation to trigeminal nucleus and the associated neurofibers could cause the release of substance-P, calcitonin gene-related peptide (CGRP) as well as other neuro-related peptide, which could lead to vasoconstriction, the origin of headaches. It had been suggested that white matter hyperintensity (WMH) in migraines might be a consequence of ischemic microvascular disturbances with prolonged and repeated oligemia during migraine attacks. The affected vulnerable minor, deep-penetrating arteries could lead to hypoperfusion in deep brain regions [16]. Some researchers confirmed that migraine patients might have hypoperfusion of cerebral blood flow [17]. Blood-brain barrier (BBB) disruption might accompany stroke, trauma, and migraine. The study suggested the possibility that peri-infarct CSD might open the BBB and promote sustained leakage of serum proteins by MMP-dependent mechanisms. Barrier disruption may contribute to cell death and an increase in infarct volume in a compromised brain [18]. Kruit and colleagues reported MRI evidence of posterior circulation territory microinfarcts in patients suffering from migraine with aura (13 of 161; 8.1%). A large number of migraine attacks predisposed individuals to ischemic lesions. Patients who had more than one attack per month reportedly had a highest risk of posterior circulation territory infarcts (odds ratio 15.8) [19]. Migraines had been consistently identified as an independent risk factor for ischemic stroke. Several potential mechanisms had been reported, including alterations in vasoreactivity and cerebral blood flow due to vessel wall dysfunction, release of vasoactive substances, platelet hyperactivity, and paradoxical embolism through a cardiac or extracardiac shunt. However, in this case, the posterior inferior cerebella artery of right side had not been blocked or straiten. Hence, the infraction in right lateral medullar might be caused by repeated attacks of VM, which could lead to cerebral vasospasm and the followed ischemia.

In this case, a 35-year-old female patient suffered from a vestibular migraine and also Wallenberg syndrome. The clinical symptoms of vertigo and migraines are in accord with the diagnostic criteria of International Headache Society (IHS) and Bárány Society, and it also conforms to the symptoms of Wallenberg syndrome. Clinically, it’s common to diagnose either vestibular migraine or Wallenberg syndrome, but it’s very rare as a concurrent disease. Due to the possibility that VM is a risk factor of cerebral ischemia, we need to pay more attention to this phenomenon. As for therapy, besides routine medication on ischemic stroke and vestibular symptom, we should also take therapy of migraine into account, such as triptans and flunarizine. It is different for the therapeutics and prophylactic criterion between this cerebral infarction and other ischemic events. A good strategy for prevention may be the key to reduce the risk of attack.

## Data Availability

The authors declare that all the data are contained within the manuscript.

## Abbreviations

VM: Vestibular migraine
MRI: Magnetic resonance imaging
PICA: Posterior inferior cerebellar artery
CSF: Cerebrospinal fluid
AQP: Aquaporin
BAEP: Brainstem auditory evoked potential
VEP: Visual evoked potential
EMG: Electromyogram
NCV: Nerve conductive velocity
DSA: Digital subtraction cerebral angiography
HIS: International Headache Society
ICHD III: International Classification of Headache Disorders 3rd Edition
fMRI: Functional magnetic resonance imaging
SSRIs: Selective serotonin reuptake inhibitors
CSD: Cortical spreading depressing
CGRP: Calcitonin gene-related peptide
